# P-50. Tigecycline for Refractory Vancomycin-Resistant Enterococcus Bacteremia

**DOI:** 10.1093/ofid/ofaf695.279

**Published:** 2026-01-11

**Authors:** Nijad Nashar, Abriana Holzworth, Matthew W Davis, Terrence McSweeney, Dayna McManus, Jeffrey E Topal

**Affiliations:** Mount Sinai Hospital, New York, NY; Yale New Haven Hospital, New Haven, Connecticut; Yale New Haven Hospital, New Haven, Connecticut; Yale New Haven Hospital, New Haven, Connecticut; Yale New Haven Hospital, New Haven, Connecticut; Yale New Haven HospitalYale University School of Medicine,, New Haven, Connecticut

## Abstract

**Background:**

Vancomycin-resistant *Enterococcus* (VRE) is associated with significant morbidity and mortality and treatment options are limited. Bacteremia refractory to daptomycin further complicates management, as linezolid use for this indication is limited by drug-related toxicities such as thrombocytopenia. Tigecycline has been explored as salvage therapy in such cases, although clinical data is limited.

**Table 1.**
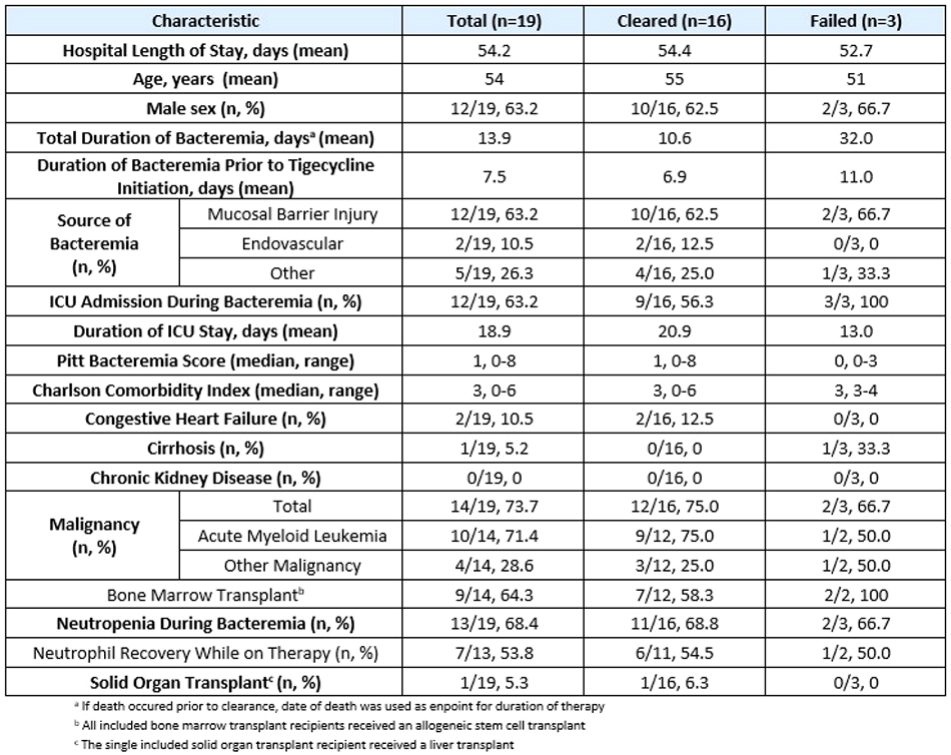
Baseline Characteristics

**Table 2.**
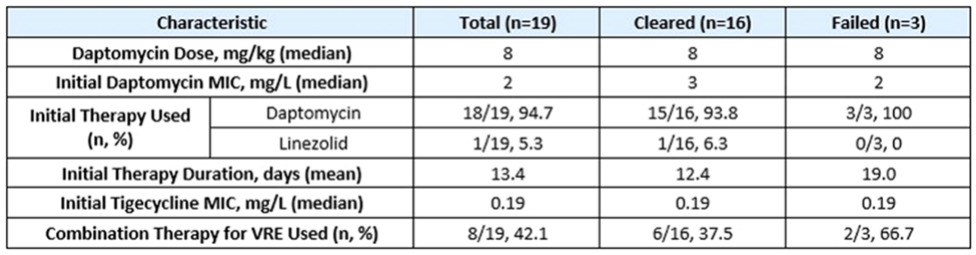
Antibiotic Therapies Administered

**Methods:**

A retrospective review was conducted on cases of refractory VRE bacteremia from January 2014 to January 2025 at Yale New Haven Hospital. Refractory bacteremia was defined as positive blood cultures for >72 hours despite empiric therapy with either daptomycin or linezolid. Patients were excluded if: age < 18 years, tigecycline was used for any indication other than VRE bacteremia, tigecycline was administered after bacteremia clearance, or if tigecycline was used as initial therapy. Patients who died prior to clearance of bacteremia were included only if tigecycline was used for >72 hours. See Table 1 for demographic data.

The primary study endpoint was clearance of bacteremia while on tigecycline. This was defined as sterilization of blood cultures while on tigecycline therapy. Secondary endpoints assessed include: time to clearance on tigecycline, in-hospital mortality, 30-day mortality, 30-day bacteremia recurrence and duration of bacteremia following tigecycline initiation. Adverse events to therapy were assessed (Table 4).

**Table 3.**
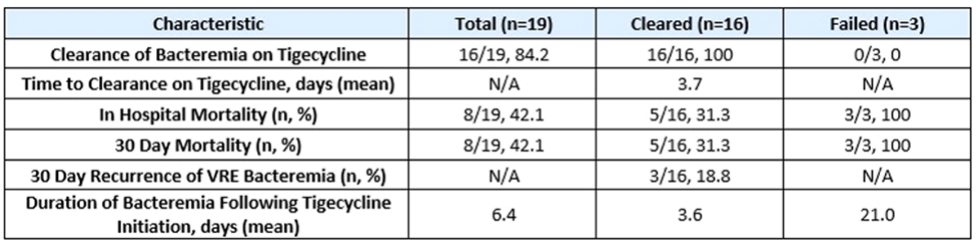
Primary and Secondary Endpoints

**Table 4.**
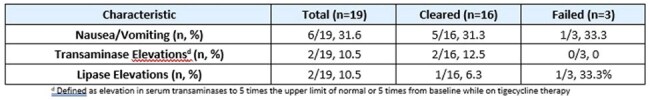
Adverse Events

**Results:**

Of the 44 cases considered, 19 met inclusion criteria. The majority of patients (74%) had a malignancy. 71% of patients with malignancy had acute myeloid leukemia and 64% received an allogeneic stem cell transplant. 68% of patients had neutropenia at the onset of VRE bacteremia. Baseline characteristics were similar between groups (Table 1). Antibiotic therapies administered are seen in Table 2.

Of the 19 cases, 84% (16/19) achieved the primary endpoint. In patients who cleared, the mean time to clearance following tigecycline initiation was 3.7 days and 30-day mortality was 31%. Safety data was favorable overall (Table 4).

**Conclusion:**

Tigecycline may be a reasonable option for the treatment of refractory VRE bacteremia, particularly in immunocompromised patient populations.

**Disclosures:**

All Authors: No reported disclosures

